# Antiobesity and Antidiabetes Effects of a* Cudrania tricuspidata* Hydrophilic Extract Presenting PTP1B Inhibitory Potential

**DOI:** 10.1155/2016/8432759

**Published:** 2016-02-18

**Authors:** Dae Hoon Kim, Sooung Lee, Youn Wook Chung, Byeong Mo Kim, Hanseul Kim, Kunhong Kim, Kyung Mi Yang

**Affiliations:** ^1^New Drug Discovery Laboratory, Hyundai Pharmaceutical Co. Ltd., Gyeong-Gi Bio-Center, Lui-dong, Yeongtong-gu, Suwon, Gyeonggi-do, Republic of Korea; ^2^Chuncheon Bioindustry Foundation, Natural Resources Commercialization Center, Chuncheon, Gangwon-do, Republic of Korea; ^3^Yonsei Cardiovascular Research Institute, Yonsei University College of Medicine, Seoul, Republic of Korea; ^4^Severance Integrative Research Institute for Cerebral & Cardiovascular Diseases (SIRIC), Yonsei University College of Medicine, Seoul, Republic of Korea; ^5^Brain Korea 21 Project for Medical Science, Yonsei University, Seoul, Republic of Korea; ^6^Department of Biochemistry and Molecular Biology, Yonsei University College of Medicine, Seoul, Republic of Korea

## Abstract

Diabetes and obesity represent the major health problems and the most age-related metabolic diseases. Protein-tyrosine phosphatase 1B (PTP1B) has emerged as an important regulator of insulin signal transduction and is regarded as a pharmaceutical target for metabolic disorders. To find novel natural materials presenting therapeutic activities against diabetes and obesity, we screened various herb extracts using a chip screening allowing the determination of PTP1B inhibitory effects of the tested compounds using insulin receptor (IR) as the substrate.* Cudrania tricuspidata* leaves (CTe) had a strong inhibitory effect on PTP1B activity and substantially inhibited fat accumulation in 3T3-L1 cells. CTe was orally administrated to diet-induced obesity (DIO) mice once daily for 3 weeks after which changes in glucose, insulin metabolism, and fat accumulation were examined. Hepatic enzyme markers (aspartate aminotransferase, AST, and alanine aminotransferase, ALT) and total fat mass and triglyceride levels decreased in CTe-treated mice, whereas body weight and total cholesterol concentration slightly decreased. CTe increased the phosphorylation of IRS-1 and Akt in liver tissue. Furthermore, CTe treatment significantly lowered blood glucose levels and improved insulin secretion in DIO mice. Our results strongly suggest that CTe may represent a promising therapeutic substance against diabetes and obesity.

## 1. Introduction

Many metabolic diseases such as diabetes, hypertension, circulatory diseases, Alzheimer's disease, and malignant cancer are associated with obesity [1–5]. Protein-tyrosine phosphatases (PTPs) are important risk factors for metabolic diseases. PTPs play an important role in maintaining the proper tyrosine phosphorylation state of proteins [6]. Abnormal tyrosine phosphorylation has been implicated in obesity and diabetes. Thus, PTP inhibitors have been investigated as potential drugs against these diseases. Protein-tyrosine phosphatase 1B (PTP1B) is a negative regulator of insulin signaling. Increased PTP1B activity results in the development of insulin resistance, subsequently leading to type 2 diabetes and obesity [7]. The insulin receptor (IR) is the most important substrate of PTP1B. On binding to its receptor, insulin induces activation of the insulin-receptor kinase (IRK) through autophosphorylation. Recruitment of insulin-receptor substrate (IRS) proteins induces activation of Akt, leading to glucose uptake in muscle [8] PTP1B dephosphorylates IR, thereby deactivating it and contributing to insulin resistance. PTP1B is also found in the brain, where it plays an important role in energy regulation by interfering with the leptin receptor, thereby, affecting the body weight status [6]. PTP1B knockout animals show increased autophosphorylation of the insulin receptor, and their myocytes and hepatocytes are more sensitive to insulin than those of wild-type animals [9, 10]. Additionally, these animals present a low body fat content and high resistance to obesity from excessive nutritional intake. PTP1B overexpression is associated with an increase in insulin levels [11]. PTP1B is considered as an attractive target for the development of new treatments for obesity and related metabolic syndromes. Although synthetic PTP1B inhibitors such as thiazolidinediones, phosphorus-containing phosphotyrosyl mimetics, dephostatin, and bidentate have been developed [12], little is known about the PTP1B inhibitory activity of drugs derived from natural resources.

The increasing incidence of obesity and diabetes is a serious socioeconomic problem in many countries. To overcome this problem, multinational pharmaceutical companies are aggressively performing studies to develop competitive drugs. Natural product-based therapeutic approaches provide a medicinal herb or fruitful source for safe, effective, and less expensive therapeutic drugs. In addition, natural products have been attracting attention because they present a multipronged mechanism of action and contain many hydrophilic active ingredients [13]. Many herbal extracts have been used for the treatment of obesity and diabetes. Extracts derived from* Peucedanum japonicum* Thunb,* Phalaris canariensis*, and* Panax ginseng* have been used to treat obesity [14–16]. Animal models and clinical studies showed that plant extracts from Apocynaceae,* Capparis spinosa*,* Indigofera spicata *Forssk, and* Antidesma bunius* possess antidiabetic effects [17–20]. Therefore, natural products are considered as promising sources for PTP1B inhibitors. Since the discovery of flavonoids as natural PTP1B inhibitors, various natural substances with an inhibitory effect on PTP activity have been reported [21–23]. Until recently, approximately 300 PTP1B inhibitors have been identified from natural resources [24]. However, further studies are needed to evaluate the effects of natural products with PTP1B inhibitory activity against diabetes and obesity.

*Cudrania (C.) tricuspidata*, a small tree of the Moraceae family, is widely used as an ingredient in oriental medicine. Several reports demonstrated that* C. tricuspidata* extract (CTe) has various effects, including inhibition of pancreatic lipase, lipopolysaccharide-induced nitric oxide production, prostaglandin E2 production in macrophages, IL-1*β*-induced rheumatoid synovial fibroblast proliferation, and matrix metalloproteinase production [25–27]. In this study, we selected a CTe from among many plant extracts using a chip screening platform that allowed us to determine PTP1B inhibitory effects of various extracts using IR as the substrate. Furthermore, we investigated the effects of CTe on metabolic disturbances associated with obesity and diabetes using a high-fat diet-induced obesity (DIO) mouse model. Our results provide a basis for developing novel antiobesity and antidiabetic medications and functional foods.

## 2. Materials and Methods

### 2.1. Extract Preparation

*C. tricuspidata* leaves were obtained from traditional Korean medical center (Goseong, Gangwon-do, Korea), which were pulverized using a blender. The pulverized leaves were subjected to reflux extraction twice using 20 times its volume of distilled water at 100°C for 8 h. The extracts were filtered using Whatman filter paper number 2 (pore size; 8 *μ*m), concentrated by using a rotary vacuum evaporator (EYELA, Tokyo, Japan), and lyophilized using a freezer dryer (PVTF20R, Ilshinbiobase, Dongduchun, Korea). CTe extraction and processing were performed in a good manufacturing practice (GMP) facility according to industry standards. The CTe yield was 28.7%.

### 2.2. PTP1B Inhibitory Assay Using a Protein Chip Screening Method

The PTP1B inhibitory activity of the tested extracts was evaluated using recombinant IR protein as a substrate. The IR was incubated at 30°C for 1 h for autophosphorylation. For the screening chip, the IR was spotted to a slide (Proteogen Co., Chuncheon, Korea) and coated overnight at 4°C. A mixture containing 0.05 *μ*g PTP1B (R&D system) and CTe was used to assess CTe inhibitory activity on PTP1B. We used orthovanadate (Na_3_VO_4_) as the positive control and 10% PEG as the negative control to evaluate the quality of our screening. The final volume of the mixture was 1 *μ*L. The mixture was added to the IR-coated slide and incubated for 1 h in a 30°C incubator. After washing, the anti-phospho-insulin-receptor antibody (used at a 1 : 100 dilution in dilution buffer; 10% bovine serum albumin and 30% glycerol in phosphate buffered saline (PBS), pH 7.4, Invitrogen, Carlsbad, CA, USA) was added and incubated in a 30°C incubator for 1 h. The secondary antibody used for visualization was an anti-rabbit IgG-Cy5 (used at a 1 : 100 dilution in dilution buffer; 10% bovine serum albumin and 30% glycerol in PBS buffer, pH 7, Invitrogen) and incubated in a 30°C incubator for 1 h. The PTP1B inhibitory activity of the extracts was visualized using a microarray scanner system (GenePix 4300A, Molecular Devices, Sunnyvale, CA, USA). The data were analyzed using GraphPad Prism4 software (GraphPad Software, San Diego, CA, USA).

### 2.3. 3T3-L1 Cell Proliferation and Differentiation

3T3-L1 preadipocytes (CL-173*™*, ATCC, Manassas, VA, USA) were cultured in Dulbecco's modified eagle medium (DMEM) medium (Sigma-Aldrich, Saint Louis, MO, USA) supplemented with 10% fetal bovine serum (FBS, Gibco Co., Carlsbad, CA, USA) and maintained at 37°C with 5% CO_2_. To induce differentiation, 3T3-L1 cells were treated with methylisobutylxanthine, dexamethasone, and insulin (MDI) induction medium (1 *μ*M dexamethasone, 0.5 mM 3-isobutyl-1-methylxanthine (IBMX) and 10 *μ*g/mL insulin in 10% DMEM medium) at approximately 100% confluence. The adipocytes were allowed to differentiate for 8 days in culture. The accumulation of lipid droplets was examined.

### 2.4. Staining with Oil Red O and Quantification

After 8 days of culture to allow cell differentiation, the culture medium was removed and the cells were fixed in 10% formalin at ambient temperature for 2 h and then washed once with 60% isopropanol. The washed cells were stained for 10 min at ambient temperature by using the Oil Red O stain (Sigma-Aldrich, St. Louis, MO, USA), which specifically reacted with lipid droplets. The cells were washed with distilled water and observed under a microscope (IX71, Olympus, Tokyo, Japan). For quantitative measurement of lipid droplets, the Oil Red O stain was extracted with 100% isopropanol and the absorbance was measured at 492 nm. The background control was 100% isopropanol.

### 2.5. Diet-Induced Obesity (DIO) Mouse Model and Treatment

The experimental animals were 4-week-old C57BL/6 mice. They were divided into the normal group and obesity/diabetes group. The animals were allowed to acclimatize for 1 week. The mice were fed a 60% high-fat diet (HFD) for 12 weeks, which increased the body weight. These animals were then used as the DIO mice. The lean group comprised C57BL/6 mice fed a normal chow. PBS was used as the placebo treatment for 3 weeks prior to treatment with CTe. At 16 weeks, the CTe treatment was conducted for 3 weeks. The CTe concentrations administered to the mice were 20 and 100 mg/kg. The effects of CTe were compared to those of sitagliptin, an antidiabetic medication, and sibutramine, an antiobesity medication, which were administered at 10 mg/kg and 5 mg/kg, respectively, to the control animals. The CTe and the drugs were administered orally once daily.

### 2.6. Measurement of Body Weight, Body Fat Content, and Plasma Lipids

After 12 weeks on the high-fat diet, mice were grouped. The mice in the vehicle group were treated with 0.5% carboxymethylcellulose solution (vehicle). The mice in the experimental groups were administered 20 or 100 mg/kg of CTe dissolved in the vehicle solution. Changes in body weight were measured for 3 weeks, every 2 days, by using an animal scale. The animals were fasted for 24 h and were then anesthetized using ether. Blood was collected from the abdominal vena cava, and the plasma was obtained by centrifugation at 3000 rpm at 4°C for 10 min. After blood sampling, the inguinal fat and epididymal fat were removed, washed with saline solution, dried, and weighed. Plasma fatty acid levels such as triglyceride (TRIG), high-density lipoprotein- (HDL-) cholesterol, and low-density lipoprotein- (LDL-) cholesterol were measured using the respective kits purchased from Roche and by using an automatic analyzer (Cobas C111, Roche, Basel, Switzerland).

### 2.7. Measurement of Serum ALT and AST Levels

Plasma alanine aminotransferase (ALT) and aspartate aminotransferase (AST) were analyzed using commercially available test kits (Thermo Scientific, Middletown, VA).

### 2.8. Measurement of Glucose and Insulin Levels

DIO mice were fasted for 1 day and orally administered with vehicle or CTe. After 30 min, a glucose bolus (2 g/kg dissolved in saline; Sigma-Aldrich) was administered to each mouse and blood was drawn from the tail vein at 0, 30, 60, 90, and 120 min after glucose administration. The blood glucose levels were measured using an Accu-Chek glucometer (Roche) and the insulin level was measured using an enzyme-linked immunosorbent assay (ELISA) kit (Abcam, Cambridge, UK).

### 2.9. Oral Glucose Tolerance Test

DIO mice were fasted for 6 h and then orally administered 20 and 100 mg/kg of CTe dissolved in 0.5% CMC. After 30 min, a glucose bolus (2 g/kg dissolved in saline; Sigma-Aldrich) was administered to each mouse. Blood glucose levels were measured at indicated time points from tail vein bleeding by Accu-Chek glucometer (Roche). The area under the curve (AUC) was calculated for each animal using the Trapezoid method.

### 2.10. Pathscan Sandwich ELISA Assay

Tissues were homogenized in RIPA buffer (50 mM Tris-HCl (pH 7.5), 150 mM NaCl, 1% NP-40, 1% sodium deoxycholate, 0.1% SDS, 2 mM EDTA, 1 mM sodium orthovanadate, 2 mM sodium pyrophosphate, and 10 *μ*g/mL leupeptin) and centrifuged to make the supernatant available for sandwich ELISA assay. The assay was carried out according to the manufacturer's instructions (Cell signaling, Danvers, MA, USA). Briefly, one hundred *μ*L of supernatant was added to each well of the anti-p-IRS-1 (panTyr) or anti-p-Akt (Ser473) coated microwell plate and then incubated for 2 h at 37°C. After being washed, the bound protein was incubated with specific antibody, followed by exposure to the HRP- (horseradish peroxidase-) linked secondary antibody and the TMB (tetramethylbenzidine) substrate. Finally, stop solution was added to each well and the absorbance at 450 nm was read by microplate reader (Molecular Device, Sunnyvale, CA, USA).

### 2.11. Statistical Analysis

Data are presented as the mean ± SD for each group. Student's* t*-test was used to evaluate statistical significance.

## 3. Results

### 3.1. Inhibitory Effects of CTe on PTP1B Activity in the Protein Chip Screening

**Figure 1:**
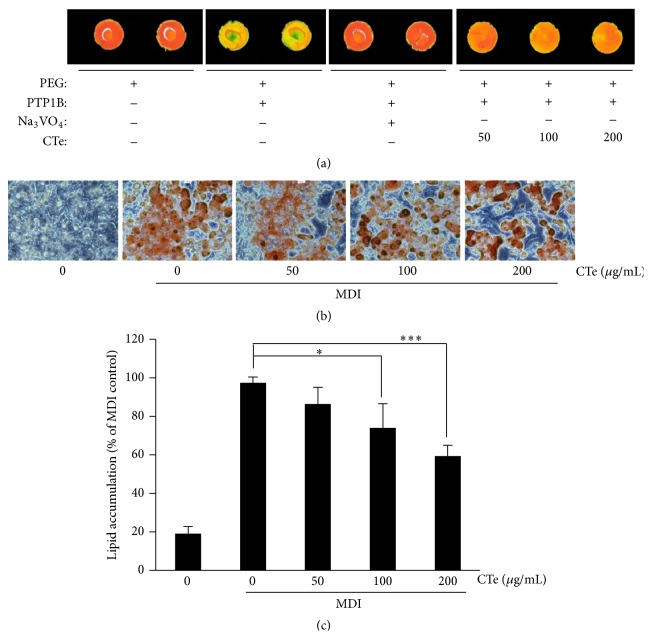
Effect of CTe on PTP1B activity and lipid droplets. (a) The substrate (IR; insulin receptor) was subjected to autophosphorylation for 1 h, followed by spotting on a chip slide. Reaction mixture was incubated for 24 h in the presence of PTP1B (100 mg/mL) with sodium orthovanadate (Na_3_VO_4_; 800 *μ*M), CTe (50, 100, or 200 *μ*g/mL), or 10% PEG alone. The fluorescent signal corresponding to the phosphorylated IR incubated with an anti-phospho-IR, followed by incubation with an anti-Cy5 antibody, was measured using a microarray scanner system. (b) CTe inhibition of fat droplet formation in 3T3-L1 cells. 3T3-L1 cells were stained with Oil Red O dye and examined using a light microscope. (c) Quantification of lipid accumulation in differentiated 3T3-L1 cells by measuring the absorbance of the cell extract at 520 nm. Statistical significance was determined by Student's *t*-test. ^*∗*^ *p* < 0.05 and ^*∗∗∗*^ *p* < 0.001 versus MDI treated cells.

The inhibitory potency of CTe on the PTP1B was evaluated with an IC_50_ value above 65 *μ*g/mL (data not shown). To identify medicinal herbs that inhibit PTP1B activity, phosphorylation inhibitory reactions were performed as described in Section 2. As shown in Figure 1(a), PTP1B potently inhibited IR phosphorylation. The CTe presented a high inhibitory activity against PTP1B similar to that of the positive control Na_3_VO_4_. The CTe contained potent inhibitors of PTP1B and displayed a high selectivity towards PTPs.

### 3.2. CTe Prevented Lipid Accumulation in 3T3-L1 Cells

To examine the effect of CTe on lipid accumulation, differentiated 3T3-L1 cells were treated with 50, 100, and 200 *μ*g/mL of CTe and the lipid droplets formed in the cells were stained with Oil Red O stain (Figures 1(b) and 1(c)). Compared to untreated differentiated cells, in which many lipid droplets were observed, CTe-treated differentiated cells presented few lipid droplets in a concentration-dependent manner (*p* < 0.05 and *p* < 0.001, resp.). Taken together, these results showed that CTe treatment inhibited lipid accumulation in 3T3-L1 cells in a dose-dependent manner.

### 3.3. Effects of CTe on the Body Weight and Body Fat Content of DIO Mice

**Figure 2:**
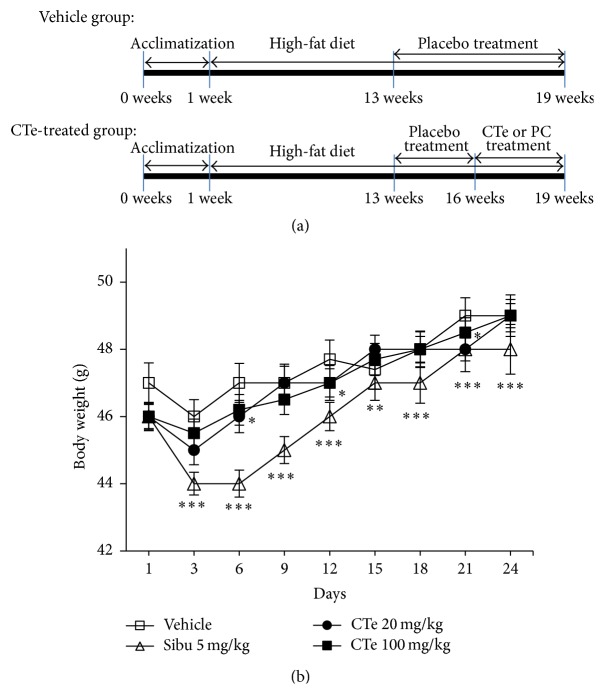
Time line of the experimental procedures using the diet-induced obesity (DIO) mouse model, and effects of CTe on the body weight of DIO mice. (a) C57BL/6 mice were treated daily with vehicle or 60% high fat diet (HFD) by oral gavage for 12 weeks. Week 0 is the starting point beginning on the 1st week with normal chow. The mice were treated with a placebo for 3 weeks prior to CTe treatment. The vehicle group received the placebo treatment for 6 weeks without oral administration of CTe (upper panel). The HFD-fed mice were orally administered CTe or a positive control (PC; sibutramine or sitagliptin) for 1 month (lower panel). (b) Body weights of DIO mice treated with vehicle or CTe (20 or 100 mg/kg) for 3 weeks. Sibutramine was used as a positive control. Data are presented as the mean ± SD for each group (*n* = 7). ^*∗*^ *p* < 0.05 and ^*∗∗∗*^ *p* < 0.001 versus vehicle administered DIO mice.

**Figure 3:**
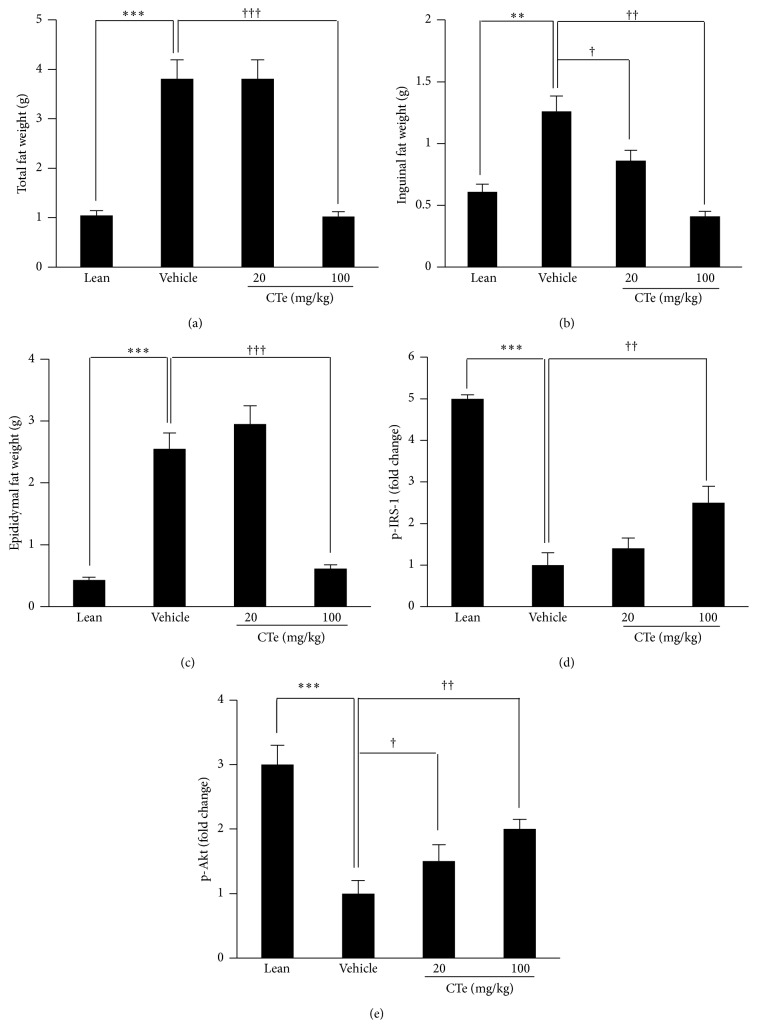
Effects of CTe on fat mass and insulin signaling in DIO mice. The HFD-fed mice were orally administered vehicle or CTe (20 or 100 mg/kg) for 3 weeks. At 28 h after the final CTe treatment, fat was collected from DIO mice. (a) Total fat weight. (b) Inguinal fat weight. (c) Epididymal fat weight. (d) p-IRS-1 and (e) p-Akt detection was estimated by Pathscan sandwich ELISA assay. Data are presented as the mean ± SD for each group (*n* = 7). Statistical significance was determined by Student's* t*-test. ^*∗∗*^ *p* < 0.01 and ^*∗∗∗*^ *p* < 0.001 versus lean mice; ^†^ *p* < 0.05, ^††^ *p* < 0.01, and ^†††^ *p* < 0.001 versus vehicle administered DIO mice.

To explore the effects of CTe on glucose tolerance and further characterize the kinetics of the effects of CTe on glucose metabolism, we generated DIO mice using C57BL/6 mice (Figure 2(a)). First, we determined the effects of CTe on weight gain in the DIO mouse model. As shown in Figure 2(b), no significant difference was observed in body weight between the vehicle- and CTe-treated groups. Consistent with previous studies, mice treated with sibutramine showed a marked decrease in body weight [28]. However, the body weight of mice treated with CTe, at both concentrations (20 and 100 mg/kg), was unchanged for up to 25 days, compared to that of the vehicle group (*n* = 7). Additionally, CTe treatment had no effect on the weights of the liver, spleen, or kidney when compared to vehicle treated-DIO mice (data not shown). Weights of the total fat (Figure 3(a)), inguinal fat (Figure 3(b)), and epididymal fat (Figure 3(c)) were significantly affected by the 100 mg/kg CTe treatment (*p* < 0.05, *p* < 0.01, and *p* < 0.001, resp.).

### 3.4. CTe Enhanced Phosphorylation of Signal Transduction Molecules in DIO Mice Liver

In order to further confirm the action mode of the CTe* in vitro*, Pathscan sandwich ELISA assay was conducted. PTP1B is involved in regulatory step of signal transduction by dephosphorylation of cellular protein; therefore, CTe probably increases phosphorylation by the inhibition of PTP1B. Pathscan sandwich ELISA assay analysis showed that CTe enhanced phosphorylation of IRS-1 and Akt signal transduction proteins in DIO mice liver (Figures 3(d) and 3(e)) (*p* < 0.05, *p* < 0.01, and *p* < 0.001, resp.). Similarly, CTe treatment increased the phosphorylation of IRS-1 and Akt in 3T3-L1 cells (data not shown). These results revealed that CTe has may be effectively contributed to the inhibition of PTP1B.

### 3.5. Effects of CTe on Plasma Lipids and Liver Enzymes

**Figure 4:**
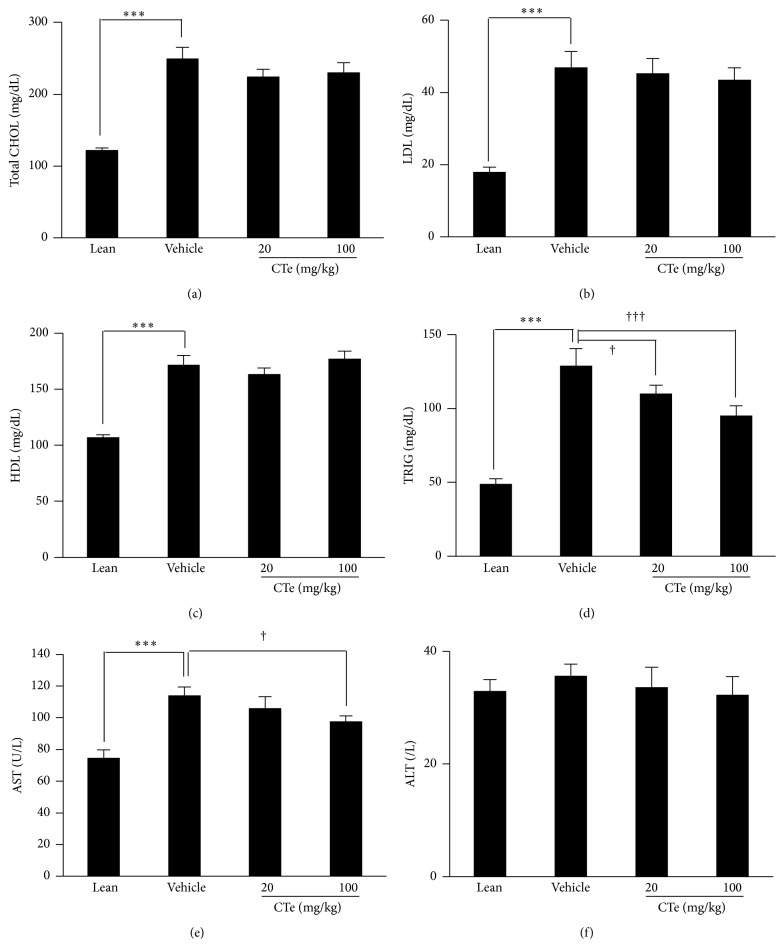
The HFD-fed mice were orally administered CTe (20 or 100 mg/kg) for 3 weeks. Blood was collected from the abdominal vein. (a) Levels of total cholesterol (CHOL), (b) low-density lipoprotein (LDL), (c) high-density lipoprotein (HDL), and (d) triglycerides (TRIG) were analyzed in triplicate using a serum biochemical analyzer. Liver tissue was collected for toxicity test. (e) Aspartate aminotransferase (AST) and (f) alanine aminotransferase (ALT) levels were monitored by clinical chemistry analyzer. Data are presented as the mean ± SD for each group (*n* = 7). Statistical significance was determined by Student's* t*-test. ^*∗∗∗*^ *p* < 0.001 versus lean mice; ^†^ *p* < 0.05 and ^†††^ *p* < 0.001 versus vehicle administered DIO mice.

Three weeks after CTe administration, we evaluated the effect of CTe on the concentration of plasma lipids. Total CHOL (Figure 4(a)) and LDL (Figure 4(b)), and HDL (Figure 4(c)) concentrations were not affected by CTe treatment. However, TRIG (Figure 4(d)) concentration was lower in the 20 or 100 mg/kg CTe-fed mice (*p* < 0.01 and *p* < 0.001, resp.). The cellular lipid accumulation in skeletal muscle has been associated with dyslipidemia and insulin resistance. Our results showed that TRIG content was significantly decreased in DIO mice by CTe treatment. In line with these findings, plasma levels of AST were lower in CTe-fed DIO mice in comparison with DIO mice fed with vehicle (Figure 4(e)) (*p* < 0.05). However, plasma ALT levels showed no differences between the two groups (Figure 4(f)).

### 3.6. Effects of CTe on Glucose and Glucose Tolerance

**Figure 5:**
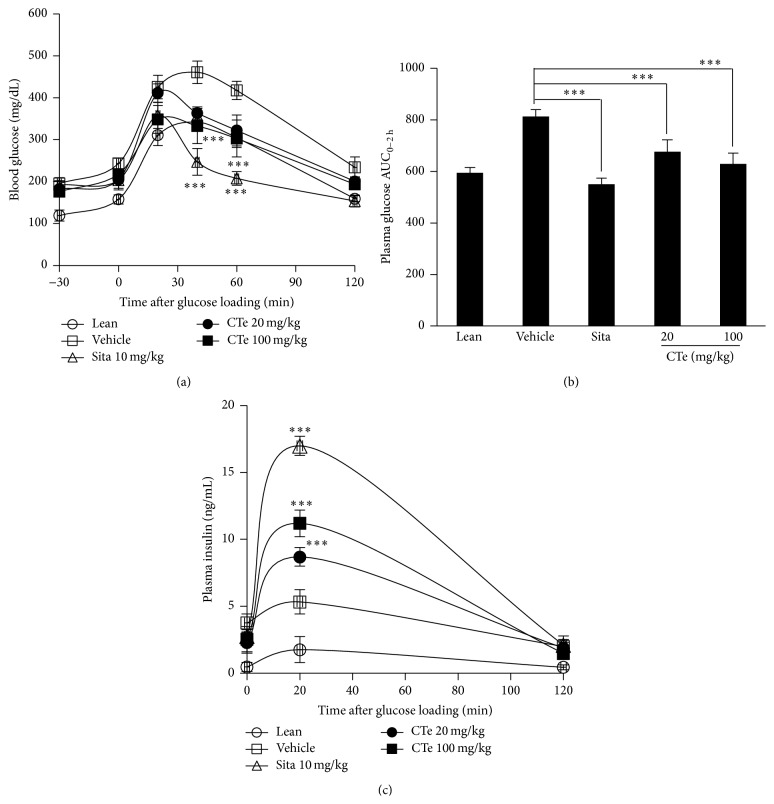
Effect of CTe on blood glucose and insulin levels in DIO mice. The HFD-fed mice were orally administered CTe (20 or 100 mg/kg) for 3 weeks. (a) The effect of CTe on plasma glucose levels in DIO mice. (b) The area under the plasma glucose concentration-time curve for 2 h (AUC_0–2 h_) in an OGTT. (c) The effect of CTe on plasma insulin in DIO mice. Sitagliptin was used as a positive control. Data are presented as the mean ± SD for each group (*n* = 7). Statistical significance was determined by Student's* t*-test. ^*∗∗∗*^ *p* < 0.001 versus vehicle administered DIO mice.

After HFD consumption, mice presented significantly elevated fasting blood glucose concentrations when compared with animals that consumed the normal diet. We next performed an oral glucose tolerance test (OGTT) to evaluate whether CTe supplementation improved the glycemic control in DIO mice. OGTT was performed on DIO mice exposed to a single oral administration of CTe at 20 and 100 mg/kg for 2 h. CTe improved glucose tolerance. In fact, significantly lower blood glucose levels at 30 and 60 min (Figure 5(a)) as well as a reduced AUC (Figure 5(b)) were observed after CTe administration following glucose bolus when compared to vehicle-treated mice. The sitagliptin-treated positive control mice showed a greater decrease in blood glucose at all time-points, and at 120 min, the blood glucose level was very low. Therefore, we concluded that CTe was effective in lowering blood glucose levels. These results revealed that CTe functions as an efficient blood glucose lowering natural product agent.

### 3.7. CTe Improved Insulin Levels

We further assessed whether CTe can prevent diabetes in DIO mice. C57BL/6 mice were fed the vehicle or a HFD containing 20 or 100 mg/kg of CTe. As shown in Figure 5(c), the level of insulin secreted 20 min after the administration of CTe was increased in the 20 and 100 mg/kg CTe groups, which were 1.6- and 2.0-fold higher, respectively, than that of the control group. The level of insulin in the sitagliptin-treated positive control group was higher than that of the CTe-treated groups. Because CTe treatment resulted in an increase in insulin in the DIO mouse model, we believe that CTe treatment could prevent diabetes.

## 4. Discussion

Obesity, a metabolic disorder concomitant with diabetes, is a serious challenge that needs to be overcome in the aging society. The recent trend of drug development is to identify plant-derived drugs effective in controlling both diabetes and obesity [7]. PTP1B is a well-established antidiabetes and antiobesity therapeutic target [24]. PTP1B plays a major role in regulating insulin signaling. Normally, insulin causes phosphorylation events through IR autophosphorylation, which enhances IR kinase activity, followed by activation of phosphatidylinositide-3-kinase (PI3K) and subsequent Akt phosphorylation cascade, which plays a central role in the control of cell growth, survival, and metabolism [29]. Mice lacking the* Ptp1b* gene presented improved insulin sensitivity with increased tyrosine phosphorylation of IR and did not develop type II diabetes or obesity. PTP1B deletion prevents myocardial anomalies in HFD-induced obesity [30]. Mice with whole body PTP1B deletion were protected against the development of obesity and diabetes. PTP1B inhibition in peripheral tissues has been useful for the treatment of metabolic syndrome and reduction of cardiovascular risks, in addition to diabetes [31]. PTP1B-directed antisense oligonucleotides are already tested in phase II clinical trials [32]. These studies indicate that PTP1B inhibitors may be promising candidates for the development of novel antidiabetic and antiobesity drugs. Natural products garnered the attention for the long-term treatment of metabolic diseases because they have a multipronged mode of action and contain multifunctional ingredients [33]. Therefore, there is a significant interest in natural products that inhibit PTP1B activity for their potential use in the treatment of obesity and diabetes.

In this study, we demonstrated that the oral administration of CTe induces protective effects against obesity and diabetic induction in the DIO mouse model. To obtain a natural substance containing active ingredients, we selected crude extracts from natural sources that have a potential inhibitory effect on PTP1B activity by monitoring their effect on protein phosphorylation levels through chip screening. Among the examined hydrophilic herb extracts, CTe had a strong inhibitory capacity towards PTP1B activity in the screening platform (Figure 1(a)). We also elucidated the antiobesity effects of CTe on fat accumulation in 3T3-L1 adipocytes by staining with Oil Red O (Figures 1(b) and 1(c)). Measurement of the blood lipid profile after oral administration of CTe showed that fat mass and TRIG were significantly decreased (Figures 3 and 4(d)) in DIO mice. Although known natural products with PTP1B inhibitory activity have been isolated and identified from various natural resources, many reagents are not effective and present poor bioavailability [24]. Our current results indicate that CTe is a potential natural product PTP1B inhibitor for the treatment of obesity and diabetes risk factors.

Some reagents showed good properties, but compared to the existing antidiabetic agents such as rosiglitazone, metformin, and pioglitazone, oral PTP1B inhibitors are difficult to design [34]. Long-term administration of nonspecific T-cell protein-tyrosine phosphatase (TC-PTP) inhibitors can be a problem. New antibody-based treatment strategies are being developed for diabetes and obesity [35]. The recently developed shRNA PTP1B inhibitor has yielded satisfactory results [7, 36]. However, studies on the development of PTP1B inhibitors are still in their early stages and the development of a safe oral substance is required.

Synthetic PTP1B inhibitors that are currently in use for the treatment of obesity have limitations because of their side effects. Moreover, their clinical application is still limited because the stability and the dosage efficacy of synthetic inhibitors have not been clearly examined. Therefore, there has been a growing interest in natural substances. CTe is an appropriate oral medication because of its high solubility. Additionally, it exhibits good activity towards PTPs* in vitro* without hepatic toxicity.* C. tricuspidata* is a well-known natural product with a variety of therapeutic effects. The chemical constitution and the pharmacological effects such as anti-inflammatory and antiproliferative action, antioxidant activities, and apoptosis induction of* C. tricuspidata* have been demonstrated [37–40]. Similarly, Kim et al. reported that the ethanol extract of* C. tricuspidata* exhibited an inhibitory effect on lipolysis activity [25]. These results show that* C. tricuspidata* is a promising antiobesity agent. Several studies reported the antiobesity, antidiabetic, and antihyperglycemic effects of various natural materials [41–43]. Meta-analysis studies have shown that supplement of natural product in human also helps improvement on glucose level and plasma lipid profile [44, 45]. The focus of many recent studies on obesity and diabetes is to determine the efficacy of natural compounds with the dual function of increasing serum insulin levels and decreasing glucose levels. Additionally, we found that CTe treatment effectively decreased blood glucose levels and increased insulin (Figure 5). The advantages of drugs derived from natural substance such as* C. tricuspidata* include fewer side effects, cost-effectiveness, and the possibility of oral administration. CTe can be used as an alternative to synthetic drugs for the treatment of diabetes. These studies showed that hydrophilic extracts containing potent PTP1B inhibitors may be promising reagents for the development of novel antidiabetic and antiobesity health enhancing drugs. Further studies should be performed to isolate the single active compound from CTe that is effective for diabetes treatment.
